# The Regimental Stretcher-Bearers

**Published:** 1915-10-16

**Authors:** 


### THE REGIMENTAL STRETCHER-BEARERS.

### Their Heroism and Grit.

An officer serving under the British Red Cross Society writes as follows:—

I was glad to see in The Hospital for October 9 the excellent summary of the duties of a medical officer attached for duty to a combatant unit. Those who read it carefully will have noted that the non-commissioned officers and men who are directly under the orders and training of a regimental medical officer as stretcher-bearers are not men of the R.A.M.C., but combatants belonging to the particular regiment or other unit to whom the medical officer is attached. These men, usually known as the regimental stretcher-bearers, are entitled to wear the Red Cross brassard, and are protected theoretically by the Geneva Convention while carrying out stretcher duties. It is not, however, of their selection or their training that I would add a word to the article in question; but rather of their work and ways.

Every officer of the R.A.M.C. at the Front whom I have come across concurs in the opinion which, from somewhat limited experience, I have formed for myself. That is, that, from a medical service point of view, the greatest devotion and heroism upon the field have been displayed by the regimental stretcher-bearers. This is not a reflection upon the officers and men of the R.A.M.C., but a simple statement of informed opinion, which is practically unanimous on the subject. The regimental stretcher-bearers, in the very nature of things, are more exposed, and more frequently exposed, to shell and rifle fire than the R.A.M.C. are; and their opportunities of displaying heroic qualities are correspondingly enlarged. If these gallant fellows have, collectively, a fault, it is an excess of courage which leads some of them to expose themselves overmuch to the enemy's fire when collecting the wounded; for, in practice, the Geneva brassard is of little use as a protection to those who wear it. It is generally recognised now, and has been for many months, that to attempt to rescue wounded in the open during daylight merely means added risk for them, and almost certain death for the rescuer. I think I run no risk of contradiction by any officer, either of the combatant services or of the R.A.M.C., when I say that the regimental stretcher-bearers have carried off the palm for valour and self-sacrificing devotion to the wounded under fire, and that they have splendidly upheld the old reputation of the British soldier for chivalry as well as for bravery in the field.

